# Malaria rapid diagnostic tests reliably detect asymptomatic *Plasmodium falciparum* infections in school-aged children that are infectious to mosquitoes

**DOI:** 10.1186/s13071-023-05761-w

**Published:** 2023-06-30

**Authors:** Lorenz M. Hofer, Prisca A. Kweyamba, Rajabu M. Sayi, Mohamed S. Chabo, Sonali L. Maitra, Sarah J. Moore, Mgeni M. Tambwe

**Affiliations:** 1 Vector Biology Unit, Department of Epidemiology and Public Health, Swiss Tropical and Public Health, Institute, Kreuzstrasse 2, 4123 Allschwil, Basel, Switzerland; 2 University of Basel, Petersplatz 1, 4001 Basel, Switzerland; 3 Vector Control Product Testing Unit (VCPTU) Ifakara Health Institute, Environmental Health, and Ecological Sciences, 74, Bagamoyo, Tanzania; 4 The Nelson Mandela African Institution of Science and Technology (NM-AIST), 447, Tengeru, Arusha, Tanzania

**Keywords:** Malaria diagnostics, *Plasmodium falciparum*, Asymptomatic malaria, Malaria transmission, *Anopheles gambiae *sensu stricto, Gametocytes

## Abstract

**Background:**

Asymptomatic malaria infections (*Plasmodium falciparum*) are common in school-aged children and represent a disease transmission reservoir as they are potentially infectious to mosquitoes. To detect and treat such infections, convenient, rapid and reliable diagnostic tools are needed. In this study, malaria rapid diagnostic tests (mRDT), light microscopy (LM) and quantitative polymerase chain reaction (qPCR) were used to evaluate their performance detecting asymptomatic malaria infections that are infectious to mosquitoes.

**Methods:**

One hundred seventy asymptomatic school-aged children (6–14 years old) from the Bagamoyo district in Tanzania were screened for *Plasmodium* spp. infections using mRDT (SD BIOLINE), LM and qPCR. In addition, gametocytes were detected using reverse transcription quantitative polymerase chain reaction (RT-qPCR) for all qPCR-positive children. Venous blood from all *P. falciparum* positive children was fed to female *Anopheles gambiae *sensu stricto mosquitoes via direct membrane feeding assays (DMFAs) after serum replacement. Mosquitoes were dissected for oocyst infections on day 8 post-infection.

**Results:**

The *P. falciparum* prevalence in study participants was 31.7% by qPCR, 18.2% by mRDT and 9.4% by LM. Approximately one-third (31.2%) of asymptomatic malaria infections were infectious to mosquitoes in DMFAs. In total, 297 infected mosquitoes were recorded after dissections, from which 94.9% (282/297) were derived from infections detected by mRDT and 5.1% (15/297) from subpatent mRDT infections.

**Conclusion:**

The mRDT can be used reliably to detect children carrying gametocyte densities sufficient to infect high numbers of mosquitoes. Subpatent mRDT infections contributed marginally to the pool of oocyts-infected mosquitoes.

**Graphical Abstract:**

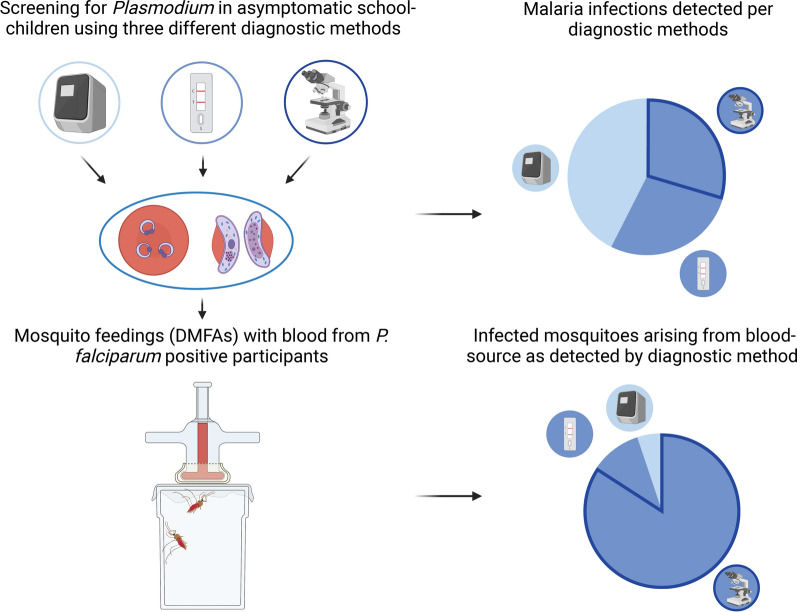

**Supplementary Information:**

The online version contains supplementary material available at 10.1186/s13071-023-05761-w.

## Introduction

Better knowledge on treatment strategies targeting the infectious reservoir of *Plasmodium falciparum* opens new avenues to tackle malaria infections that are asymptomatic and infectious to mosquitoes [1]. Asymptomatic malaria infections are common among school-aged children (6–15 years old) [2] and are characterised by higher complexity of infection [3, 4] and higher gametocyte prevalence than those with symptomatic infections [5]. Some proportions of school-aged children with asymptomatic malaria infections are particularly infectious to mosquitoes [6, 7] and consequently serve as a reservoir for *Plasmodium* transmission due to persistent asymptomatic infections [5, 8]. A mass test-and-treat (MTAT) approach for school-aged children may be a promising tool to target asymptomatic *Plasmodium* infections and reduce the transmission reservoir [9, 10], especially as school-aged children tend to be underserved by current control tools such as insecticide-treated nets (ITNs) [11].

In previous studies, mRDTs have been used for detection of asymptomatic malaria infections. For example, in community studies, a limited reduction in malaria transmission was reported from Zambia [12] while in Burkina Faso [13] no significant reductions in clinical malaria cases were observed. In Kenya, screening and treatment of school-aged children had no impact on their health or educational performance [14]. One possible explanation was the limited sensitivity of standard mRDTs in detecting low-density asymptomatic infections suggesting the need for using molecular techniques for future interventions [15, 16]. On the other hand, school-based interventions using standard mRDTs in Malawi found that MTAT reduced gametocyte prevalence and density [10].

Low-density asymptomatic infections—including those with gametocyte densities potentially infectious to mosquitoes—are often undetected by LM and mRDT, the standard diagnostic tools in malaria diagnostics. When compared to more sensitive molecular tools such as qPCR, LM and mRDT have repeatedly shown inferior performance [16–18]. In general, the likelihood of a mosquito becoming infected is dependent on gametocyte density as well as on male-to-female sex ratio of gametocytes [19]. Nonetheless, microscopic and submicroscopic gametocyte densities do infect mosquitoes [20, 21], with the latter being rarely detected by mRDTs. The question remains whether malaria infections subpatent to commercially available mRDTs contribute substantially to malaria transmission especially as low-density infections are unlikely to infect mosquitoes [5, 16].

Here, we investigated the diagnostic performance of a commercially available mRDT (SD BIOLINE) compared with LM and qPCR for detecting infectious and asymptomatic malaria infections among school-aged children. To confirm onward transmission to mosquitoes DMFAs were performed. Our aim was to investigate whether parasite densities subpatent to mRDTs are infectious to mosquitoes in DMFAs. Using qPCR and RT-qPCR, we measured the infectiousness of microscopic and submicroscopic gametocyte densities from humans to mosquitoes.

## Methods

### Study design, participants and ethics

**Fig. 1:**
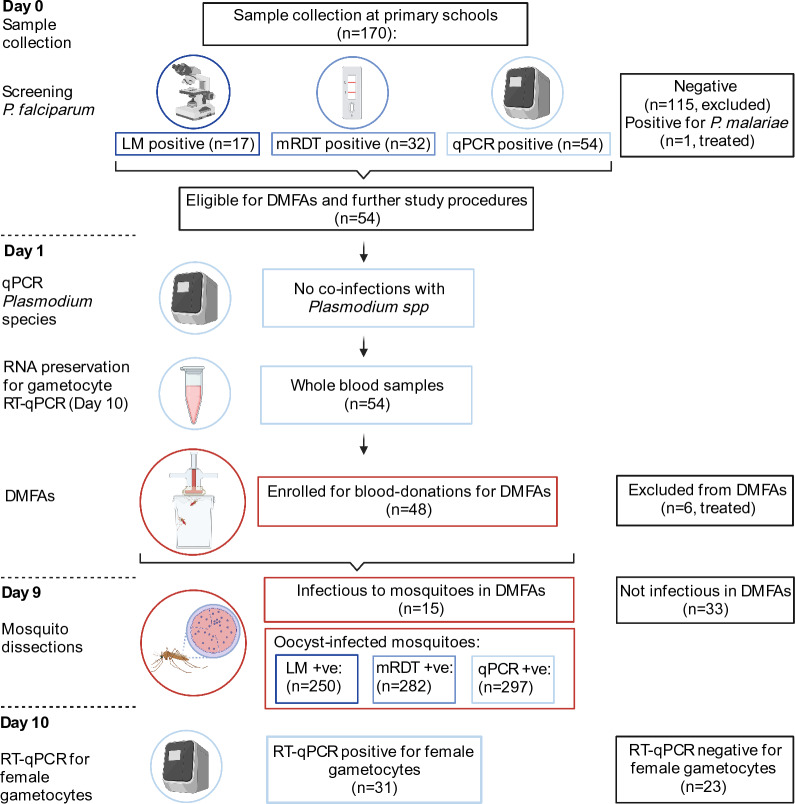
Overview of the study procedures. Participants were screened systematically for *Plasmodium* infections using qPCR, mRDT, LM and RT-qPCR. All *Plasmodium falciparum* qPCR-positive children were invited to participate in DMFAs. Infectiousness of children participating in DMFAs was assessed by mosquito dissection and oocyst detection by microscopy 8 days post-infection. Numbers of infected mosquitoes were attributed to the diagnostic method, which detected the infectious participants

This cross-sectional study was conducted in two primary schools of Buma and Yombo located in the Bagamoyo district in coastal Tanzania. After the long rainy season that lasts from March to June, we recruited 170 asymptomatic school-aged children over a period of 10 weeks between June and the end of August 2020. Children aged 6–14 years were eligible for enrolment when written informed consent from their caregivers and assent from the children was obtained. All children screened positive for *Plasmodium* infection were enrolled for blood-drawing for DMFAs and then treated with artemether-lumefantrine (ALU). The treatment was done within 24 h of diagnosis according to national guidelines in Tanzania [22]. An overview of all study procedures is given in Fig. 1.

### Sample preservation for nucleic acid extraction

All participants were finger pricked using a safety lancet (MiniCollect® Safety-Lancets 2 mm, Greiner, Austria) to obtain a capillary blood sample into an EDTA containing collection tube (Mini Collect® Tube 0.25/0.5 ml K3EDTA, Greiner, Austria). Then, 180 µL whole blood was transferred into Eppendorf tubes containing 540 µL of 1 × DNA/RNA Shield™ (Zymo Research, Irvine, USA) for nucleic acid preservation until extraction of DNA (same day of preservation).

### Malaria rapid diagnostic test (mRDT)

Examination of the presence of *Plasmodium* using mRDT (SD BIOLINE Malaria Ag P.f./Pan (HRPII/pLDH), Standard Diagnostic, South Korea) was executed according to the manufacturer’s instructions. The mRDT detects the *P. falciparum*-specific histidine-rich protein 2 (HRPII) antigen targeting the parasites asexual blood stages and *Plasmodium* species in general via the parasite lactate dehydrogenase (pLDH), including sexual stages (gametocytes). One drop (~ 50 µL) of capillary blood was applied to the test and run with four drops of a delivered standard buffer. The test was kept on a flat surface while running and read by the laboratory technician after 15 min. The result was confirmed and recorded by a second member of the field team trained on reading the mRDT.

### Light microscopy (LM)

Thick smears were prepared using capillary blood from the finger prick. Dried thick smears were stained for 45 min with 10% Giemsa stain and examined for the presence of *Plasmodium* parasites under a light microscope (100 × magnification, oil immersion). Slides were considered negative if no *Plasmodium* parasites were detected after examination of 100 consecutive fields. After detection of the first parasites, asexual parasites (ring stages) were counted per 200 white blood cells (WBCs) and gametocytes were counted per 500 WBCs.

### Molecular detection and quantification of malaria infections by qPCR

The DNA for molecular diagnosis and quantification of *Plasmodium* infections was manually extracted from 180 µL whole blood preserved as described above using the Quick-DNA™ Miniprep kit (Zymo Research) according to the manufacturer’s instructions. DNA was eluted in 50 µL elution buffer and stored at − 20 °C.

*Plasmodium falciparum* infections were detected and quantified using the PlasQ qPCR assay [23, 24]. To quantify parasite blood-stage parasites, the WHO international standard for *P. falciparum* quantification [23] was used. Serial dilutions ranging from 100,000 parasites/µL—0.0001 parasites/µL were prepared and run in triplicate. All primers and probes are listed in Additional file 1: Table S1. Amplification and qPCR measurements were performed using the Bio-Rad CFX96 Real-Time PCR System (Bio-Rad Laboratories, USA). The thermal profile used for the assay was as follows: 60 s at 95 °C; 45 cycles of 15 s at 95 °C and 45 s at 57 °C. Each reaction contained 2 µL DNA and 8 µL reaction master mix containing 1 × Luna Universal Probe qPCR Master Mix (New England Biolabs, USA). All qPCR assays were run in duplicate with appropriate controls including Non-Template Control (NTC) and *P. falciparum* NF54 DNA as positive control (PC).

To investigate *P. falciparum* co-infections with other relevant parasite species in the Bagamoyo district [25, 26], all *Plasmodium*-positive samples were subjected to further analysis using the PlasID qPCR assay as previously described [27]. Samples were analysed with reagents, volumes and instruments as described above. Appropriate controls for all *Plasmodium* species were included. The thermal profile used for the assay was as follows: 5 min at 95 °C; 45 cycles of 15 s at 95 °C and 60 s at 55 °C.

### Molecular detection of female gametocytes by RT-qPCR

Samples for ribonucleic acid (RNA) extraction were taken from all qPCR-positive (PlasQ assay) children and 150 µL of whole blood was transferred into an Eppendorf tube containing 450 µL 1 × DNA/RNA Shield™. The tubes were mixed by inversion, spun down for 30 s and incubated for 60 min at room temperature before subsequent storage at − 20 °C until further use. RNA was manually extracted using the Quick-RNA™ MiniPrep Plus kit (Zymo Research). The protocol was adapted as per manufacturer’s suggestions: (i) after thawing of frozen samples, 100 µL of 1 × DNA/RNA Shield™ was added and vortexed for 60 s at full speed; (ii) Proteinase K digestion was extended to 60 min at room temperature; (iii) after Proteinase K digestion, the samples were centrifuged for 5 min at 16,000 × rcf to pellet all debris of red blood cells (RBCs). On-column DNase I digestion of gDNA was performed on all samples according to the manufacturer’s instructions [28]. RNA was eluted in 50 µL of prewarmed (95 °C) DNase/RNase-free water and stored at − 80 °C until further use.

Female gametocytes were detected using a RT-qPCR assay designed by Wampfler et al. [29, 30]. Reverse transcription, amplification and qPCR measurements were performed using the same instrument as above with the following thermal profile: 10 min at 50 °C; 60 s at 95 °C; 40 cycles of 10 s 95 °C and 60 s at 58 °C. Each reaction contained 4 µL RNA and 10 µL reaction master mix containing Luna Universal 1 × One-Step RT-qPCR Kit (New England Biolabs, USA).

### Mosquito rearing

We used the *Anopheles gambiae *sensu stricto (Ifakara strain) colony, which is reared at IHI, Bagamoyo Branch Insectary, Kingani. The colony is maintained under standard conditions at a 12:12 h dark:light cycle, 70 ± 20% humidity and 27 ± 2 °C. Larvae are fed on TetraMin® fish flakes (Tetra Ltd., UK) and reared at a density of 200 larvae (from L2 larval stage) per 1 L deionized water. Adult mosquitoes are kept in 30 × 30 × 30 cm containers and allowed to feed on 10% sucrose solution. Adult mosquitoes are routinely checked for microsporidia infections [31, 32]. Cages with microsporidia-infected mosquitoes are discarded and strict hygiene measures are maintained in the insectary.

### Direct membrane feeding assays and dissection of mosquitoes

Unfed female mosquitoes (3–5 days old, mated) were sugar starved overnight with access to water only. The next morning, a batch of 50 female mosquitoes was transferred into paper cups. For each participant, six cups were prepared. Blood from *P. falciparum*-positive, asymptomatic school-aged children was drawn for DMFAs in lithium-heparin-coated vacutainers. Whole blood was transferred in pre-warmed 1.5 mL Eppendorf tubes centrifuged for 3 min at 300 rcf and 37 °C [33]. Autologous serum was removed and replaced with pre-warmed malaria-naïve AB serum [34] (37 °C). Mosquitoes were fed from water-jacketed glass feeders (39 °C) covered with a Parafilm® membrane. Each feeder was filled with 200 µL of blood and mosquitoes were allowed to feed for 10–15 min in the dark [35]. After the infectious blood meal, mosquitoes were kept at 75 ± 2% humidity and 27 ± 1 °C at 12:12 h dark:light cycle in a climatic chamber (AraLab, Portugal). The next morning, unfed mosquitoes were removed and allowed to feed on 10% sucrose solution. Eight days post-infection, mosquitoes were dissected and midguts stained for 10 min using 1% mercurochrome solution (Sigma, Switzerland) before examination under a compound microscope at 40 × magnification for the presence of oocysts.

### Statistical analysis

Data was entered in Excel (Microsoft, 2016) and analysed using the statistical software R and the R studio (Version 1.3.1093) interface. Sensitivity of each diagnostic test was manually calculated using 2 × 2 contingency tables. Proportions of positive and negative events of diagnostic tests including 95% confidence intervals (CIs) were calculated by one-sample z-tests. Positive predictive values of diagnostic tests in predicting infected mosquitoes deriving from infectious asymptomatic individuals were calculated as percentages of infected mosquitoes of the respective diagnostic tool, divided by all infected mosquitoes detected with qPCR.

## Results

### Study population

A total of 170 children participated in the study from Buma (*N* = 80) and Yombo (*N* = 90) primary schools. Mean age among study participants was 10.3 years and participants were 64% female (*N* = 109) and 36% male (*N* = 61).

### Malaria prevalence detected by molecular and standard diagnostic tools

**Fig. 2:**
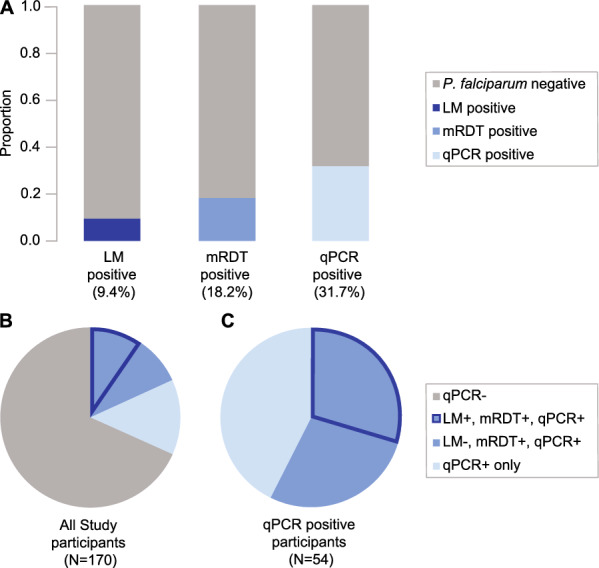
Malaria prevalence detected separately by each of the three diagnostic methods **A**; LM (9.4%), mRDT (18.2%) and qPCR (31.7%). Proportions **B** of all study participants (*N* = 170) either negative (68.3%, grey) or as detected by all three methods together (9.4%, dark blue margin), by mRDT and qPCR but not LM (8.8%, medium blue) or qPCR only (13.5%, light blue). Proportions **C** of all qPCR-positive participants (*N* = 54) as detected by all three methods together (29.6%, dark blue margin), by mRDT and qPCR but not LM (27.8%, medium blue) or qPCR only (42.6%, light blue). *LM* light microscopy; *mRDT* malaria rapid diagnostic test; *qPCR* quantitative polymerase chain reaction

We screened 170 asymptomatic school-aged children for malaria infection using three different diagnostic methods (qPCR, mRDT, LM). Of 170, 54 were qPCR-positive for *P. falciparum* infection (31.7%, PlasQ assay, Fig. 2A) and one was positive for *Plasmodium malariae* (0.6%, PlasID assay) infection. No mixed infections were detected. Asexual blood-stage densities of *P. falciparum* molecularly quantified by qPCR ranged from 0.1 parasites/µL to 275,000 parasites/µL with a median parasite density of 6 parasites/µL. About 18.2% of study participants were mRDT positive (Fig. 2A) with median parasite density of 35 parasites/µL as quantified by qPCR and 9.4% were LM positive (Fig. 2A) with a median parasite density of 1493 parasites/µL. Approximately eighteen percent (18.2%) of the study participants were tested positive on gametocyte carriage using RT-qPCR and 2.3% of all study participants had microscopically detectable gametocytes. Gametocyte densities as quantified by LM ranged from 16 gametocytes/µL to 128 gametocytes/µL, with median gametocyte density of 48 gametocytes/µL. All LM-positive participants were also positive for mRDT and qPCR. These results are summarized in Table 1. Sensitivity of mRDT and LM compared to qPCR was 56.3% and 30.9% respectively and both methods showed > 99% specificity (Table 2).

**Table 1:** *Plasmodium* infections in 170 asymptomatic school-aged children detected by qPCR assays, mRDT and LM

Diagnostics	*n*^a^	*n*/*N*^b^ (%)^c^	95% CI (%)^d^	Parasite densities per diagnostic method
qPCR				
*P. falciparum *(PlasQ assay)	54	31.7	25.2–39.1	6 (0.1–275,000)
*P. malariae *(PlasID assay)	1	0.6	0.001–3.3	–
mRDT	31	18.2	13.2–24.7	35 (0.1–275,000)^e^
LM				
*P. falciparum* all stages	16	9.4	5.9–14.7	1439 (133–173,000)^f^
*P. falciparum* asexual stages	10	5.8	3.2–10.5	–
*P. falciparum* gametocytes	6	2.3	0.1–5.9	48 (16–128)^g^
*P. malariae *all stages	1	0.6	0.001–3.3	–
Gametocyte-specific RT-qPCR				
*P. falciparum* female gametocytes (pfs25)	31	18.2	13.2–24.7	–

*qPCR* quantitative polymerase chain technique; *mRDT* malaria rapid diagnostic test; *LM* light microscopy; *RT-qPCR* reverse transcriptase quantitative polymerase chain technique

^a^*n* = number of positive participants

^b^*N* = 170 participants tested in total

^c^n/N (%) = percent positive

^d^95% CI =  95% confidence interval

^e^Median parasite densities quantified by PlasQ as detected by qPCR and mRDT in parasites/µL. Range of densities in brackets

^f^Median asexual parasite densities quantified by LM in parasites/µL. Range of densities in brackets

^g^Median sexual parasite densities quantified by LM, in gametocytes/µL. Range of densities in brackets

**Table 2:** Sensitivity and specificity of mRDT and LM compared to qPCR in 170 study participants

Diagnostics	qPCR positive (*n* = 55)	qPCR negative (*n* = 115)	Sensitivity % (95% CI)	Specificity % (95% CI)
mRDT positive (*n* = 32)	31	1	56.3	99.1
mRDT negative (*n* = 138)	24	114		
LM positive (*n* = 17)	17	0	30.9	100
LM negative (*n* = 153)	38	115		

*qPCR* quantitative polymerase chain reaction; *mRDT* malaria rapid diagnostic test; *LM* light microscopy

### Infectiousness of asymptomatic individuals through DMFAs relative to detection by molecular or standard diagnostic tools

**Fig. 3:**
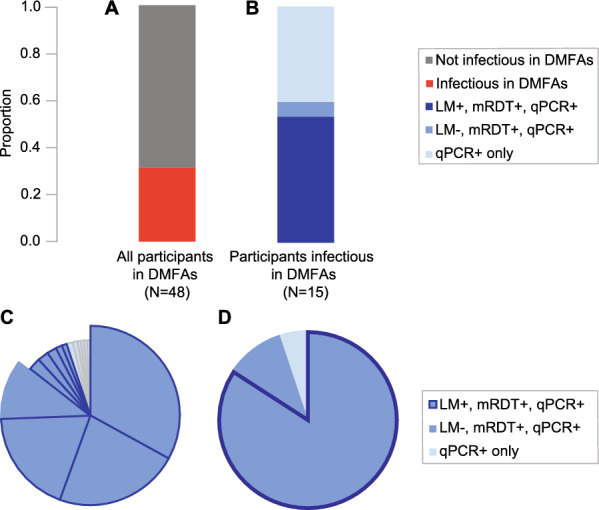
**A** Proportion of study participants infectious to at least one mosquito in DMFAs (15/48, 31.2%) from all qPCR-positive study participants enrolled for DMFAs (*N* = 48). **B** Participants infectious to mosquitoes (*N* = 15) as detected with all three methods (eight participants, dark blue margin), mRDT and qPCR but not LM (nine participants, medium blue) and qPCR only (six participants, light blue). **C** Oocyst-infected mosquitoes per participant, majority of mosquitoes infected by only four participants (big slices, 85%). **D** Oocyst-infected mosquitoes from blood sources as detected with all three methods (84.2%, *N* = 250, dark blue margin), mRDT and qPCR positive but not LM (94.9%, *N* = 282 medium blue) and qPCR only (5.1%,* N *= 15 light blue). *LM* light microscopy; *mRDT* malaria rapid diagnostic test; *qPCR* quantitative polymerase chain reaction; *DMFA* direct membrane feeding assay

Of the 54 qPCR (PlasQ) *P. falciparum*-positive children, six were not enrolled for DMFAs and were treated directly with ALU. Reasons for exclusion were developing symptoms (*n* = 2), withdrawn from the study by caregiver (*n* = 1), fear of needle pricking for blood drawing (*n* = 2) and insufficient mosquito numbers for DMFAs (*n* = 1). The remaining 48 qPCR-positive children were enrolled for DMFAs. From those, 15 (31.2%, Fig. 3A) were infectious to mosquitoes and infected at least one mosquito. Results of DMFAs are summarized in Table 3. In total, we dissected 5022 mosquitoes on day 8 post-infection with an average of 105 dissected mosquitoes per DMFA. We recorded 297 oocyst-infected mosquitoes from the 15 infectious DMFAs with median oocyst intensity of two oocysts per infected mosquito. The highest oocyst number in a single midgut reached 29. Only four participants infected 253 out of 297 oocyst-infected mosquitos (85%, Fig. 3C). The remaining 15% of oocyst-infected mosquitoes were infected from 11 infectious participants.

**Table 3:** Results of DMFAs and positive predictive value for oocyst-infected mosquitoes per diagnostic method

Diagnostics	DMFAs resulting in mosquito infection^a^		Proportion of oocyst-infected mosquitoes^b^		Positive predictive value^c^
	*n/N*	% (95% CI)	*n/N*	% (95% CI)	% (*n*/qPCR)
qPCR					
*Plasmodium falciparum* (PlasQ)	15/48	31.2 (19.9–45.3)	297/1610	18.4 (16.6–20.4)	100 (297/297)
mRDT					
*P. falciparum*	9/31	29.0 (16.1–46.5)	282/1005	28.1 (25.3–30.9)	95.0 (282/297)
LM					
*P. falciparum* asexuals only	3/10	30 (10.8–60.3)	18/391	4.6 (2.9–7.1)	6.1 (18/297)
*P. falciparum* gametocytes	5/6	83.3 (43.6–96.9)	232/643	36.0 (32.5–39.9)	78.1 (232/297)
*P. falciparum* all stages	8/16	50 (27.9–72)	250/930	26.8 (24.0–29.8)	84.2 (250/297)
RT-qPCR					
*P. falciparum* female gametocytes (pfs25)	15/31	48.4 (32.0–65.1)	297/1610	18.4 (16.6–20.4)	100 (297/297)

*qPCR* quantitative polymerase chain reaction; *mRDT* malaria rapid diagnostic test; *LM* light microscopy; *RT-qPCR* reverse transcription quantitative polymerase chain reaction; *DMFA* direct membrane feeding assay

^a^All 48 participants enrolled for DMFAs were qPCR positive for *P. falciparum*; n/N is the proportion of DMFAs resulting in at least one infected mosquito (*n*) out of all infections detected by the corresponding diagnostic method (*N*)

^b^Proportion of mosquitoes scored positive by microscopy for oocysts on day 8 post-infection (*n*) out of total mosquitoes dissected of all DMFAs that resulted in at least one infected mosquito with the corresponding diagnostic method (*N*)

^c^Positive predictive value for infected mosquitoes by diagnostic method compared to qPCR, which predicted 100% of all infected mosquitoes resulting from DMFAs

All infected mosquitoes derived from infectious participants detected by qPCR and RT-qPCR. (Table 3 and Fig. 3B). Nine participants detected by mRDT infected 94.9% (282) of all infected mosquitoes. Consequently, subpatent mRDT infections accounted for 5.1% (15) of all infected mosquitoes only. Eight participants detected by LM-infected 84.2% (250) of all infected mosquitoes. This also represents the contributions of microscopic (84.2%) and sub-microscopic (15.8%) infections from human to mosquito transmission as recorded by this study (Fig. 3D).

## Discussion

The aim of this study was to compare the performance of standard diagnostic tools (mRDT and LM) to a sensitive molecular tool (qPCR) in detecting asymptomatic malaria infections that are infectious to mosquitoes. To our knowledge, this is the first study that specifically assesses the performance of an mRDT including the onward transmission of detected infections to mosquitoes using DMFAs.

We observed that nearly one-third (31.2%, 15/48, Fig. 3A) of all asymptomatic malaria infections were infectious to mosquitoes in DMFAs. Of those, 60% were detected by mRDT and 53% by LM (Fig. 3B). The finding that most asymptomatic *P. falciparum* infections that are infectious to mosquitoes are detected by standard diagnostic tools is consistent with evidence from other African countries [5, 6, 36]. When comparing the performance of the mRDT and LM regarding infectiousness to mosquitoes, only one infectious participant was detected by mRDT but subpatent to microscopy (Fig. 3C), suggesting that with improved microscopy (e.g. using a second microscopist for proof-reading) the performance of mRDT and LM would be nearly equal. Of all infected mosquitoes, 95% derived from infections that were detected by mRDT and 85% of infections detected by LM. A small proportion of infected mosquitoes (5%) derived from infections subpatent to both standard diagnostic tools and was detected by qPCR only.

Furthermore, we report that 85% of all infected mosquitoes derived from only four children; all of them were detected by mRDT and three were LM-positive for gametocytes (Fig. 3C). Our finding that a very small proportion of our study population accounts for the vast majority of mosquito infections supports recent findings from Uganda [5]. Also, exclusively from these four participants, we observed mosquitoes with higher oocyst densities that are more likely to develop higher densities of sporozoites [37]. These mosquitoes remain infectious for a longer time than their counterparts with mostly one or rarely two oocysts per midgut, as observed in mosquito infections from only qPCR-positive participants [19].

We have demonstrated that mRDTs identified a slightly higher number of the qPCR-positive infections compared to LM. While LM performance may be further improved by including a second microscopist, the success of the mRDT was encouraging and indicates its potential to be used alone for detection of infectious asymptomatic individuals. The advantage of using mRDTs over LM is that it is convenient and does not need highly skilled experts to interpret the results. Our findings on sensitivity and specificity concur with a previous study that evaluated the same mRDT product in Tanzania [38]. Also similar differences in sensitivity between LM (30.9%) and qPCR (100%) as reported here have been reported from low transmission settings before [39]. The proportion of subpatent infections in standard diagnostic tools that are only detected with molecular assays is very similar to previous findings from a low-transmission area in coastal Kenya [6].

The reported malaria prevalence places the Bagamoyo district as a moderate transmission area [40], similar to other coastal regions in East Africa, e.g. in Kenya [6]. Results presented in this study are in line with broad scientific consensus regarding the performance of all three diagnostic methods in asymptomatic malaria infections as reported elsewhere [20, 41, 42].

In our study, a small proportion of children was LM positive for gametocytes, but when applying molecular diagnostics, we found that nearly every fifth child carried female gametocytes as detected by RT-qPCR. These results were not surprising and comparable to gametocyte prevalence detected by LM and RT-qPCR in previous studies from Tanzania [2, 43] and other low and moderate transmission areas in sub-Saharan Africa [6, 36, 42]. Results presented here are limited because we have not screened for male gametocytes or quantified both female and male gametocytes using molecular methods.

Measuring the transmissibility of malaria infections using DMFAs has several limitations [34]. Here, we conducted DMFAs with serum replacement to remove transmission-blocking attributes and to increase the transmission potential of parasite isolates in the blood samples fed to mosquitoes [44]. Thereby, loss of infectiousness of gametocytes when fed through membranes [45] can be partly compensated. We observed mostly low intensities of oocyst infections with median oocyst density of two in infected midguts. This is what is commonly observed in natural infections and xenodiagnostic surveys [46]. While oocyst detection is regarded as standard end point for measuring parasite transmission in membrane feeding assays (MFAs) [46], dissection for oocysts does not reflect the true onward-transmission potential of mosquitoes. Including the detection of sporozoites would help to better understand the infectiousness of mosquitoes.

## Conclusion

Commercially available mRDTs may be a suitable and convenient tool to identify asymptomatic malaria infections that are infectious to mosquitoes. Although mRDTs failed to detect a substantial number of asymptomatic malaria infections compared to the total infections detected by qPCR, the mRDT detected infections that accounted for 95% of all oocyst-infected mosquitoes after DMFAs. Many low-density infections that are subpatent to mRDTs could become infectious to mosquitoes at a later time point. Therefore, repeated testing with mRDTs and treatment may be required if implemented as an intervention to reduce the reservoir of transmission and numbers of infectious mosquitoes in a community efficiently [5, 47]. Finally, as our study was comparatively small, we would recommend testing larger cohorts of school-aged children in additional settings to increase statistical power and if possible include more sensitive mRDTs for the evaluation of diagnostic tools [48].

## Supplementary Information

**Additional file 1:** Oligos used in this study.

**Additional file 2:** Complete data set of the present study.

## Data Availability

All data generated or analysed during this study are included in this published article as Additional file 2.

## Abbreviations

mRDT: Malaria rapid diagnostic test
qPCR: Quantitative polymerase chain reaction
RT-qPCR: Reverse transcription quantitative polymerase chain reaction
LM: Light microscopy
DMFA: Direct membrane feeding assay
MTAT: Mass test and treat
IPT: Intermittent preventive treatment
ALU: Artemether–lumefantrine
DNA: Deoxyribonucleic acid
HRPII: Histidine-rich protein 2
pLDH: Parasite lactate dehydrogenase
WBC: White blood cell
RBC: Red blood cell
RNA: Ribonucleic acid
18 s rDNA: *Pan-plasmodium* 18S ribosomal DNA sequence
VarATS: *P. falciparum*-specific acidic terminal sequence of the var genes
IHI: Ifakara Health Institute
NIBSC: National Institute for Biological Standards and Control
CI: Confidence interval
