# Supplementary material for: Malaria rapid diagnostic tests reliably detect asymptomatic Plasmodium falciparum infections in school-aged children that are infectious to mosquitoes
Source: Parasit Vectors. 2023 Jun 30;16:217. doi: 10.1186/s13071-023-05761-w (PMC10314504; doi:10.1186/s13071-023-05761-w)
Supplement: Supplementary file 1 — Additional file 1: Oligos used in this study. [file 13071_2023_5761_MOESM1_ESM.docx]

Additional file 1. Oligos used in this study.

| **Oligo name** | **Species specificity** | **Target region** | **Oligo sequence** | **Oligo modification [5’-3’]** | **Concentration 5x Oligo Mix** | **Adapted from** |
| --- | --- | --- | --- | --- | --- | --- |
|  |  |  |  |  |  |  |
| **a) *P. falciparum* (PlasQ assay)** |  |  |  |  |  |  |
|  |  |  |  |  |  |  |
| Pspp18S fwd | *Plasmodium spp* | 18S rDNA | GCT CTT TCT TGA TTT CTT GGA TG | - | 2 µM | Schindler et al, 2019 |
| Pspp18S rev | *Plasmodium spp* | 18S rDNA | AGC AGG TTA AGA TCT CG TTC G | - | 2 µM | Schindler et al, 2019 |
| Pspp18S probe | *Plasmodium spp* | 18S rDNA | ATG GCC GTT TTT AGT TCG TG | Cy5-BHQ2 | 1 µM | Schindler et al, 2019 |
| HsRNaseP fwd | *H. sapiens* | RnaseP gene | AGA TTT GGA CCT GCG AGC G | - | 1 µM | Schindler et al, 2019 |
| HsRNaseP rev | *H. sapiens* | RnaseP gene | GAG CGG CTG TCT CCA CAA GT | - | 1 µM | Schindler et al, 2019 |
| HsRNaseP probe | *H. sapiens* | RnaseP gene | TTC TGA CCT GAA GGC TCT GCG CG | YakimaYellow-BHQ1 | 0.5 µM | Schindler et al, 2019 |
| PfvarATS fwd | *P. falciparum* | varATS | CCC ATA CAC AAC CAA YTG GA | - | 1 µM | Schindler et al, 2019 |
| PfvarATS rev | *P. falciparum* | varATS | TTC GCA CAT ATC TCT ATG TCT ATC T | - | 1 µM | Schindler et al, 2019 |
| PfvarATS probe | *P. falciparum* | varATS | TRT TCC ATA AAT GGT | FAM-NFQ/MGB | 0.5 µM | Schindler et al, 2019 |
|  |  |  |  |  |  |  |
| **b) *Plasmodium* all species (PlasID assay)** |  |  |  |  |  |  |
|  |  |  |  |  |  |  |
| PfvarATS fwd | *P. falciparum* | varATS | CCC ATA CAC AAC CAA YTG GA | - | 2 µM | Schindler et al, 2019 |
| PfvarATS rev | *P. falciparum* | varATS | TTC GCA CAT ATC TCT ATG TCT ATC T | - | 2 µM | Schindler et al, 2019 |
| PfvarATS probe | *P. falciparum* | varATS | TRT TCC ATA AAT GGT | FAM-NFQ/MGB | 1.25 µM | Schindler et al, 2019 |
| PmPlasp4 fwd | *P. malariae* | Plasp4 gene | CCA ACA ATA CAT ACA CAT TAG AAC C | - | 2 µM | Schindler et al, 2019 |
| PmPlasp4 rev | *P. malariae* | Plasp4 gene | GTA GGA TAT AAA GCA TAC ACA AAG TG | - | 2 µM | Schindler et al, 2019 |
| PmPlasp4 probe | *P. malariae* | Plasp4 gene | ATC TAG TAA TGG CTC C | YakimaYellow-BHQ1 | 1.25 µM | Schindler et al, 2019 |
| PoRBP2 fwd | *P. ovale* | Rpb2 gene | CCA CAG ATA AGA AGT CTC AAG TAC GAT ATT | - | 2 µM | Schindler et al, 2019 |
| PoRBP2 rev | *P. ovale* | Rpb2 gene | TTG GAG CAC TTT TGT TTG CAA | - | 2 µM | Schindler et al, 2019 |
| PoRBP2 probe | *P. ovale* | Rpb2 gene | TGA ATT GCT AAG CGA TAT C | TexasRed-BHQ2 | 1.25 µM | Schindler et al, 2019 |
|  |  |  |  |  |  |  |
| **c) Gametocytes** |  |  |  |  |  |  |
| Pfs25 fwd | *P. falciparum* | Pfs25 gene | GAA ATC CCG TTT CAT ACG CTT G | - | 3.57 µM | Wampfler et al, 2013 |
| Pfs25 rev | *P. falciparum* | Pfs25 gene | AGT TTT AAC AGG ATT GCT TGT ATC TAA | - | 3.57 µM | Wampfler et al, 2013 |
| Pfs25 probe | *P. falciparum* | Pfs25 gene | TGT AAG AAT GTA ACT TGT GGT AAC GGT | HEX-BHQ1 | 1.25 µM | Wampfler et al, 2013 |
|  |  |  |  |  |  |  |
|  |  |  |  |  |  |  |
